# An Endogenous Quantum–Classical Crossover Temperature in the van der Waals Fluid: Quantumness as an Emergent Behavior

**DOI:** 10.3390/e28070789

**Published:** 2026-07-12

**Authors:** Flavia Pennini, Angelo Plastino

**Affiliations:** 1 Departamento de Física, Universidad Católica del Norte, Av. Angamos 0610, Antofagasta 1270709, Chile; 2 Departamento de Física, Facultad de Ingeniería, Universidad Nacional de Mar del Plata (UNMDP), CONICET, Av. J.B. Justo 4302, Mar del Plata 7600, Argentina; 3 Instituto de Física La Plata—CCT-CONICET, Universidad Nacional de La Plata, C.C. 727, La Plata 1900, Argentina; angeloplastino@gmail.com

**Keywords:** van der Waals gas, classical–quantum crossover, grand-canonical ensemble, thermal de Broglie wavelength

## Abstract

The onset of quantum behavior in gases is traditionally established through a criterion that is external to classical statistical mechanics. One introduces the thermal de Broglie wavelength and compares it with the mean intermolecular separation, concluding that quantum effects become relevant when $n\lambda_{T}^{3}∼1$. This condition originates in quantum statistical mechanics and is absent from the classical ideal-gas or van der Waals partition functions. In this work, we show that a grand-canonical treatment of the van der Waals fluid naturally generates an interaction-corrected crossover temperature $T_{3}\left(a,b,m,n\right)$ determined by the particle mass, density, and van der Waals interaction parameters. While the thermal de Broglie wavelength provides the standard quantum crossover scale, the interaction-induced correction leading to $T_{3}$ is obtained without invoking the explicit form of the Bose–Einstein or Fermi–Dirac distributions. Instead, $T_{3}$ follows from a self-consistent condition within the grand-canonical van der Waals description. We demonstrate that, below this temperature, the statistical assumptions underlying the classical theory become self-inconsistent, indicating the breakdown of the classical description and the onset of the quantum-degenerate regime. The resulting temperature scale therefore provides an interaction-corrected boundary of validity of the classical van der Waals description. These findings provide a new perspective on how intermolecular interactions modify the crossover to the quantum-degenerate regime and clarify the limits of applicability of the classical van der Waals theory.

## 1. Introduction

The question of how and when a classical many-particle system ceases to admit a purely classical description remains one of the central issues of statistical physics. For dilute gases, classical thermodynamics and kinetic theory provide an accurate account of equilibrium and transport phenomena over broad ranges of density and temperature. However, as the temperature decreases or the density increases, quantum effects eventually become unavoidable, requiring the replacement of Maxwell–Boltzmann statistics by Bose–Einstein or Fermi–Dirac statistics [1,2].

In conventional treatments, the onset of quantum behavior is determined through a criterion that is introduced from outside classical statistical mechanics. One compares the thermal de Broglie wavelength [1,2,3]

(1) $$\lambda_{T}=\frac{h}{\sqrt{2\pi mk_{B}T}},$$

with the mean intermolecular spacing $n^{-1/3}$, where *n* denotes the particle density. Quantum effects become significant when [1,2]

(2) $$n\lambda_{T}^{3}∼1.$$

This condition follows naturally from quantum statistical mechanics and expresses the overlap of neighboring wave packets [2,4]. Importantly, however, it does not arise from the classical partition function itself. Historical attempts to incorporate such quantum corrections directly into classical phase-space frameworks have traditionally relied on expansions in powers of $\hbar^{2}$ or quantum cluster methods [5,6,7]. The standard criterion remains externally imposed and serves as an indicator of the regime in which the assumptions of classical statistics fail.

The van der Waals equation of state occupies a distinguished place in statistical physics as the simplest thermodynamic model capable of incorporating both intermolecular attraction and excluded-volume effects [8,9,10,11]. Despite its classical origin, the model successfully captures essential features of real fluids, including the existence of a liquid–gas phase transition and a critical point. In recent years, renewed attention has been devoted to the statistical foundations of the van der Waals fluid, particularly within the grand-canonical framework, where fluctuations and probability distributions can be analyzed explicitly [12,13]. Although the van der Waals equation is traditionally regarded as a purely classical model, its parameters encode microscopic information about intermolecular forces and excluded-volume effects. One may therefore ask whether the statistical structure of the theory contains intrinsic signatures of its own domain of applicability. In particular, it is natural to investigate whether, once the standard thermal-wavelength criterion has been adopted, a characteristic crossover temperature scale can be determined self-consistently within the grand-canonical formalism without making explicit use of the Bose–Einstein or Fermi–Dirac distribution functions. This perspective is complementary to foundational approaches to the quantum-to-classical transition, which typically analyze the problem from the opposite direction—investigating the emergence of classicality from a quantum substrate via decoherence or semiclassical limits [14,15,16,17].

In this work we show that the grand-canonical van der Waals fluid contains an unexpected feature. The present treatment predicts an interaction-corrected crossover temperature

(3) $$T_{3}=T_{3}\left(a,b,m,n\right),$$

which depends on the van der Waals parameters *a* and *b*, the particle mass *m*, and the density *n*. This temperature reduces to the conventional ideal-gas crossover temperature in the dilute limit while incorporating interaction effects at finite density [18,19]. Although the thermal de Broglie wavelength provides the standard quantum crossover scale, the interaction-induced correction leading to $T_{3}$ is obtained without making explicit use of the Bose–Einstein or Fermi–Dirac distribution functions [20,21].

Our central observation is that $T_{3}$ can be interpreted as an interaction-corrected boundary of validity of the classical van der Waals description. Above this temperature, the statistical assumptions underlying the model remain self-consistent, whereas, below it, the classical description is expected to lose its statistical self-consistency. The grand-canonical formulation therefore identifies the temperature range over which the classical van der Waals description is expected to remain valid [22].

This perspective complements the conventional thermal-wavelength criterion. Rather than replacing the standard condition for quantum degeneracy, the present approach shows how intermolecular interactions modify it through a self-consistent grand-canonical treatment of the van der Waals fluid. The resulting temperature $T_{3}$ therefore provides an interaction-corrected boundary of validity of the classical van der Waals description and establishes a direct connection between intermolecular interactions, excluded-volume effects, and the onset of the quantum-degenerate regime.

The purpose of the present paper is to derive this interaction-corrected crossover temperature within the grand-canonical van der Waals framework, analyze its dependence on the microscopic parameters of the fluid, and discuss its physical interpretation as an interaction-corrected boundary of validity of the classical van der Waals description.

The paper is organized as follows. In Section 2, we introduce the theoretical framework within the grand-canonical ensemble and review the conventional criterion for the classical–quantum crossover in dilute gases. In Section 3, we develop a self-consistent mean-field treatment and derive the interaction-corrected energy per particle. Section 4 is devoted to the analysis of the corresponding crossover temperature, including its representation in reduced variables and the emergence of a universal fixed point. In Section 5, we discuss the interpretation of the crossover temperature as an interaction-corrected boundary of validity of the classical van der Waals description. The low-density limit and its physical implications are examined in Section 6. Finally, Section 7 summarizes the main results and presents our concluding remarks.

## 2. Theoretical Framework

### 2.1. Diluted Interacting Gas

We consider a dilute gas of *N* identical particles interacting through a pair potential, contained in a volume *V* in equilibrium at temperature *T*. The canonical partition function is given by [1]

(4) $$Q_{N}\left(V,T\right)=\frac{1}{N!}{\left(\frac{V}{\lambda_{T}^{3}}\right)}^{N}\;Z_{N}\left(V,T\right),$$

with $\beta =1/k_{B}T$ ($k_{B}$ the Boltzmann constant), and $Z_{N}\left(V,T\right)$ is the configurational integral [1,13], which is given by

(5) $$Z_{N}\left(V,T\right)=\frac{1}{V^{N}}\;\int d^{3N}r\;\exp\left(-\beta \sum_{i<j}u_{ij}\right),$$

where $u_{ij}\equiv u\left(r_{ij}\right)$ denotes the pair interaction potential between particles *i* and *j*, with $r_{ij}=\left|r_{i}-r_{j}\right|$ the distance between them. The normalization ensures that $Z_{N}\to 1$ in the ideal-gas limit.

In order to proceed analytically within the grand-canonical ensemble, we further introduce a mean-field closure in which the instantaneous density N/V is replaced by its average value n=〈N〉/V [1,13]. This yields the approximate form

(6) $$Z_{N}\left(V,T\right)\approx \exp\left(-NnB_{2}\left(T\right)\right),$$

which captures interaction effects at the level of the second virial coefficient while allowing for a closed-form evaluation of Ξ. This approximation amounts to replacing the fluctuating particle density by its mean value.

The grand partition function of the system is defined as [1]

(7) $$\Xi =\sum_{N=0}^{\infty }\;z^{N}Q_{N}\left(V,T\right),$$

with $z=\exp(\mu /k_{B}T)$ the fugacity. Within the present approximation, one obtains

(8) $$\ln\Xi \left(\beta ,z\right)=\frac{zV}{\lambda_{T}^{3}}\exp\left[-n\left(\beta ,z\right)\;B_{2}\left(\beta \right)\right].$$

The mean energy is given by the standard grand-canonical relation [1]

(9) $$U=-{\left(\frac{\partial \ln\Xi }{\partial \beta }\right)}_{z,V},$$

which, in view of Equation (8), becomes

(10) $$U=\langle N\rangle \left(\frac{3}{2\beta }+nB_{2}^{\prime }\right),$$

with the notation $B_{2}^{\prime }=dB_{2}/d\beta$.

The chemical potential of the system is [1]

(11) $$\mu =k_{B}T\ln\left[n\lambda_{T}^{3}\;\exp\left(nB_{2}\left(T\right)\right)\right].$$

For a van der Waals gas, the interaction potential is typically modeled as a hard-core repulsion plus an attractive term at longer ranges. One can approximate the second virial coefficient $B_{2}$ as [1,13]

(12) $$B_{2}\left(T\right)=b-\beta a,$$

with $b=2\pi r_{0}^{3}/3$ being linked to the volume of a hard-sphere gas (excluded volume per molecule due to the finite size of the particles) [13], whose mean potential energy reads [1]

(13) $$a=2\pi \int_{r_{0}}^{\infty }\;dr\;r^{2}u\left(r\right).$$

For a dilute gas, with nb≪1 (low density), one has the celebrated van der Waals equation of state [1,13]

(14) $$P=\frac{k_{B}Tn}{1-bn}-an^{2},$$

and the mean energy per particle u=U/〈N〉 is given by [1,13]

(15) $$u=u_{0}-na,$$

with $u_{0}=3/\left(2\beta \right)$ being the energy per particle of the classical ideal gas [1].

### 2.2. Classical–Quantum Crossover

We start our analysis considering the classical ideal gas to recapitulate ideas about the classical–quantum crossover. From a quantum viewpoint, the single-particle energy levels in this system are very close to one another and the energy spectrum is divided into a large number of groups of levels [1]. The average energy of a level is denoted by ε. Thus, if μ is the chemical potential of the system, one expects classical Maxwell–Boltzmann behavior whenever [1]

(16) $$\exp\;\left[\beta (\varepsilon -\mu )\right]\gg 1.$$

Approximating the characteristic single-particle energy by the classical mean kinetic energy,

(17) $$\varepsilon_{0}=\frac{U_{0}}{N}=\frac{3}{2}k_{B}T,$$

the classical regime is obtained whenever [18,19,23,24]

(18) $$\exp\;\left(\beta \mu \right)\ll \exp\;\left(\frac{3}{2}\right).$$

The classical–quantum crossover in dilute gases is usually characterized by the condition

(19) $$n\lambda_{T}^{3}\ll \exp\;\left(\frac{3}{2}\right),$$

or, equivalently, by taking into account the definition of the thermal wavelength, the system remains in the classical regime for temperatures $T\gg T_{0}$, where

(20) $$T_{0}=\frac{2\pi \hbar^{2}}{m\;k_{B}\;e}\;n^{2/3},$$

defines the crossover temperature [18,19,23,24].

Extending the preceding considerations to a dilute van der Waals gas, characterized by the parameters *a* (attraction) and *b* (excluded volume), with nb≪1 and $B_{2}=b-\beta a$, and taking again $\varepsilon =U/N=\varepsilon_{0}-na$, we now have

(21) $$\exp\left(\beta \mu \right)\ll \exp\;\left(\beta \varepsilon \right),$$

which is satisfied if the ratio nb remains very small as the vdW gas becomes similar to the ideal one. Using $\varepsilon =\varepsilon_{0}-na$ together with $\varepsilon_{0}=3k_{B}T/2$, one obtains

(22) $$\exp\left(\beta \mu \right)\ll \exp\left(\frac{3}{2}-\beta an\right).$$

Substituting Equation (11) together with $B_{2}=b-\beta a$, we find

(23) $$n\lambda_{T}^{3}\;\exp\left(nb-\beta an\right)\ll \exp\left(\frac{3}{2}-\beta an\right),$$

so that the attractive contribution cancels out identically, yielding

(24) $$n\lambda_{T}^{3}\ll \exp\left(\frac{3}{2}-nb\right).$$

Alternatively, this condition can be contextualized by expressing Equation (21) directly in terms of the second virial coefficient $B_{2}\left(T\right)$,

(25) $$n\lambda_{T}^{3}\;\exp\left[nB_{2}\left(T\right)\right]\ll \exp\left(\beta \varepsilon \right),$$

implying that, as expected from the cancellation of the attractive term,

(26) $$n\lambda_{T}^{3}\ll \exp\left(\frac{3}{2}-nb\right).$$

Calling $T_{2}$ the lower bound for the validity of these classical considerations, the system remains classical for temperatures $T\gg T_{2}$, where

(27) $$T_{2}=\frac{2\pi \hbar^{2}}{m\;k_{B}\;e}\;n^{2/3}\;\exp\;\left(\frac{2nb}{3}\right)$$

defines the modified crossover temperature. Taking into account Equation (20), this expression can also be written as

(28) $$T_{2}=T_{0}\;\exp\;\left(\frac{2nb}{3}\right).$$

This result shows that, at this level of approximation, the crossover boundary is independent of attractive interactions and depends exclusively on excluded-volume effects due to the finite size of the particles. With the purpose of incorporating attractive interactions in a self-consistent manner, we now consider the possibility that *n* might depend implicitly on β.

## 3. Self-Consistent Mean-Field Correction

The previous treatment neglects the implicit temperature dependence induced by a self-consistent density relation. We now incorporate this effect explicitly [25].

The average particle number is obtained from

(29) $$\langle N\rangle =z{\left(\frac{\partial \ln\Xi }{\partial z}\right)}_{\beta ,V}=\frac{zV}{\lambda^{3}}\exp\left[-n\left(\beta ,z\right)\;B_{2}\left(\beta \right)\right],$$

which yields the implicit equation for the density

(30) $$n\left(\beta ,z\right)=\frac{z}{\lambda^{3}}\exp\left[-n\left(\beta ,z\right)\;B_{2}\left(\beta \right)\right].$$

Equation (30) defines *n* as a self-consistent function of (β,z). This structure is typical of mean-field descriptions of interacting systems and does not introduce ambiguity, provided the implicit dependence is handled consistently. Using Equation (30), one can rewrite

(31) lnΞ=Vn(β,z),

which holds identically within the present mean-field approximation [1,13].

The mean energy is given by the standard grand-canonical relation (9) [1]. Since n=n(β,z) depends implicitly on β, the derivative must be computed consistently. Using Equation (30), one obtains

(32) $$U=-V{\left(\frac{\partial n}{\partial \beta }\right)}_{z}.$$

Differentiating Equation (30) with respect to β at fixed *z*, and defining $B_{2}^{\prime }\equiv dB_{2}/d\beta$, we find

(33) $$\left(1+nB_{2}\left(\beta \right)\right){\left(\frac{\partial n}{\partial \beta }\right)}_{z}=-n\left(\frac{3}{2\beta }+nB_{2}^{\prime }\right),$$

which leads to the final expression

(34) $$U=Vn\;\frac{\left(\frac{3}{2\beta }+nB_{2}^{\prime }\right)}{1+n\;B_{2}},$$

with $B_{2}$ given by Equation (12), where *a* characterizes attractive interactions and *b* represents excluded-volume effects.

The energy per particle becomes

(35) $$u_{3}=\frac{U}{N}=\frac{\frac{3}{2\beta }-na}{1+nb-\beta an}.$$

Substituting into Equation (24) yields

(36) $$n\lambda_{T}^{3}=\exp\left(\frac{3-2\beta na}{2(1+nb-\beta an)}\right).$$

**Figure 1:**
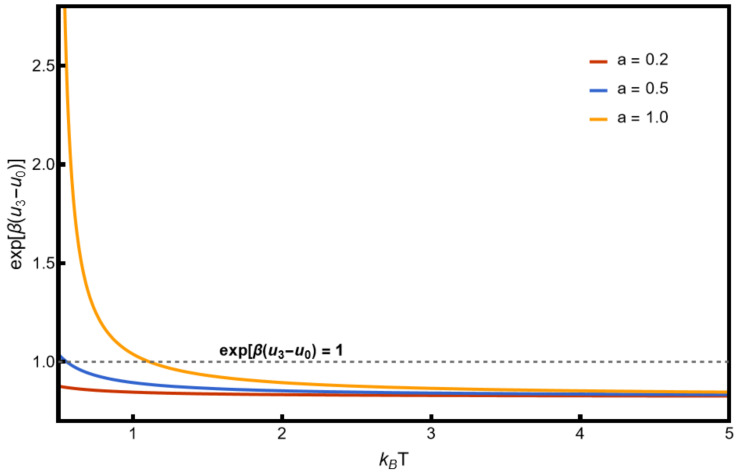
Interaction-induced correction factor $\exp[\beta \left(u_{3}-u_{0}\right)]$ as a function of $k_{B}T$ for n=0.5, b=0.3, and attractive interaction strengths a=0.2, 0.5, and 1.0. The dashed line marks the ideal-gas value $\exp[\beta \left(u_{3}-u_{0}\right)]=1$. Values above (below) unity indicate an enhancement (reduction) of the effective quantum-degeneracy criterion relative to the ideal gas. The influence of attractive interactions is most pronounced at low temperatures and becomes progressively weaker as the temperature increases, where all curves converge toward the ideal-gas limit.

In Figure 1 we plot the quantities $\exp(-\beta u_{0})$ and $\exp(-\beta u_{3})$ as functions of temperature, where $u_{0}$ denotes the ideal-gas energy per particle and $u_{3}$ the interaction-corrected result obtained within the self-consistent mean-field approach. The figure illustrates how interactions modify the classical–quantum crossover condition through a displacement of the intersection region between both curves.

The logarithmic representation is particularly useful because it explicitly separates the linear excluded-volume contribution from the sub-linear attractive correction.

## 4. Transition Temperature

Using $\lambda_{T}^{3}={\left(2\pi \hbar^{2}/mk_{B}T\right)}^{3/2}$, Equation (36) becomes

(37) $$n{\left(\frac{2\pi \hbar^{2}}{mk_{B}T}\right)}^{3/2}=\exp\left(\frac{3-2\beta na}{2(1+nb-\beta an)}\right).$$

Equation (36) defines the interaction-corrected crossover temperature $T_{3}$ through a self-consistent condition since $\beta ={\left(k_{B}T_{3}\right)}^{-1}$. An equivalent implicit representation is obtained by expressing the thermal wavelength explicitly and raising both sides to the power 2/3, leading to

(38) $$T_{3}=\frac{2\pi \hbar^{2}}{mk_{B}}n^{2/3}\exp\left(-\frac{1-2\beta na/3}{1+nb-\beta an}\right).$$

Equation (38) is mathematically equivalent to Equation (36) and makes explicit how the ideal-gas crossover temperature is modified by the van der Waals interaction parameters through an interaction-dependent exponential factor. Since $\beta ={\left(k_{B}T_{3}\right)}^{-1}$, Equation (38) remains an implicit self-consistent equation for $T_{3}$.

In addition, Equation (38) can be written in terms of the uncorrected crossover temperature $T_{0}$, given by Equation (20), as follows:

(39) $$T_{3}=T_{0}\exp\left(\frac{nb-\beta na/3}{1+nb-\beta an}\right).$$

As an illustration, we solve the self-consistent Equation (38) for liquid ^4^He, one of the prototypical quantum fluids, whose low atomic mass makes quantum effects significant at experimentally accessible temperatures [26]. We employ the standard van der Waals parameters for helium, $a=3.46\times 10^{-3}\;\mathrm{Pa}\;m^{6}\;{\mathrm{mol}}^{-2}$ and $b=2.38\times 10^{-5}\;m^{3}\;{\mathrm{mol}}^{-1}$, together with the liquid helium density $\rho =125\;\mathrm{kg}\;m^{-3}$. The resulting crossover temperature is

(40) $$T_{3}∼2.63\;K.$$

This value is remarkably close to the temperature range where quantum effects become dominant in ^4^He. In particular, it is of the same order as the superfluid transition temperature $T_{\lambda }=2.17\;K$, although it should not be interpreted as a prediction of the superfluid transition itself. Rather, $T_{3}$ identifies the onset of the regime where the classical van der Waals description is expected to lose validity and quantum statistical effects become significant. The appearance of superfluidity requires additional microscopic physics beyond the scope of the present classical thermodynamic approach.

As a representative example, we evaluate the crossover temperature for ^4^He using the experimental van der Waals parameters and the liquid density $\rho =125\;\mathrm{kg}\;m^{-3}$. The resulting value, $T_{3}=2.63\;K$, is obtained by solving the self-consistent Equation (38). Table 1 compares this temperature with other characteristic temperature scales for helium.

The ordering of the characteristic temperatures,

(41) $$T_{\lambda }<T_{3}<T_{\mathrm{BEC}}<T_{0},$$

is physically meaningful. The ideal-gas estimate $T_{0}$ provides the highest crossover temperature because it neglects intermolecular interactions. Incorporating the van der Waals corrections lowers the crossover temperature to $T_{3}$, bringing it closer to the temperature range where quantum effects become important in ^4^He. Although $T_{3}$ does not predict the onset of superfluidity, its position between the superfluid transition temperature $T_{\lambda }$ and the ideal Bose–Einstein condensation temperature $T_{\mathrm{BEC}}$ supports its interpretation as a characteristic temperature marking the breakdown of the classical description and the onset of the quantum-degenerate regime.

### 4.1. Temperature-Independent Fixed Density

An important property of Equation (39) can be identified directly in the original variables without introducing reduced quantities. Writing Equation (39) as

(42) $$\frac{T_{3}}{T_{0}}=\exp\;\left(\frac{n\left(b-\beta a/3\right)}{1+n\left(b-\beta a\right)}\right),$$

and defining the variable x=βa, the exponent becomes

(43) $$F\left(n,x\right)=\frac{n(b-x/3)}{1+n(b-x)}.$$

A density at which the temperature ratio becomes independent of temperature is obtained from the condition

(44) $$\frac{\partial F}{\partial x}=0.$$

A straightforward calculation yields

(45) $$\frac{\partial F}{\partial x}=\frac{-n+2n^{2}b}{3{\left[1+n(b-x)\right]}^{2}},$$

so that the temperature-independent solution is

(46) $$n^{*}=\frac{1}{2b}.$$

Substituting Equation (46) into Equation (42), one finds

(47) $$F\left(n^{*},x\right)=\frac{1}{3},$$

independently of temperature. Consequently, we find that

(48) $${\left.\frac{T_{3}}{T_{0}}\right|}_{n=n^{*}}=e^{1/3}≃1.3956.$$

Therefore, the theory predicts the existence of an intrinsic fixed-density point, determined solely by the excluded-volume parameter *b*, at which the crossover-temperature ratio becomes completely independent of temperature. This result emerges directly from the self-consistent mean-field structure and does not require the introduction of critical variables.

### 4.2. Reduced-Variable Representation

The fixed-density point obtained above is an intrinsic property of the self-consistent crossover relation and follows directly from Equation (42). For comparison with the conventional van der Waals corresponding-state framework, it is useful to rewrite the theory in terms of the standard reduced variables

(49) $$n_{r}=\frac{n}{n_{c}},\;T_{r}=\frac{T}{T_{c}},$$

with

(50) $$n_{c}=\frac{1}{3b},\;T_{c}=\frac{8a}{27bk_{B}}.$$

The intrinsic fixed density given by Equation (46) then becomes

(51) $$n_{r}^{*}=\frac{n^{*}}{n_{c}}=\frac{3}{2}.$$

Thus, the universal intersection observed in the reduced representation corresponds to the reduced image of the physical density $n^{*}=1/\left(2b\right)$.

### 4.3. Universal Fixed Point in Reduced Variables

Using

(52) $$nb=\frac{n_{r}}{3},\;\beta an=\frac{9}{8}\frac{n_{r}}{T_{r}},$$

Equation (39) becomes

(53) $$\frac{T_{3}}{T_{0}}=\exp\left(\frac{\frac{n_{r}}{3}-\frac{3}{8}\frac{n_{r}}{T_{r}}}{1+\frac{n_{r}}{3}-\frac{9}{8}\frac{n_{r}}{T_{r}}}\right).$$

Equation (53) is expressed entirely in terms of the dimensionless variables $n_{r}$ and $T_{r}$, making it independent of the specific microscopic parameters of the fluid. From Equation (46), the temperature-independent density $n^{*}=1/\left(2b\right)$ corresponds to $n_{r}^{*}=\frac{3}{2}$. Substituting this value into Equation (53) immediately yields

(54) $${\left.\frac{T_{3}}{T_{0}}\right|}_{n_{r}=3/2}=e^{1/3}≃1.3956.$$

Therefore, all reduced isotherms intersect at the universal fixed point

(55) $$n_{r}^{*}=\frac{3}{2},\;{\left(\frac{T_{3}}{T_{0}}\right)}^{*}=e^{1/3}≃1.3956.$$

This result is simply the reduced-variable representation of the intrinsic fixed-density point $n^{*}=1/\left(2b\right)$ derived above.

### 4.4. Physical Interpretation

**Figure 2:**
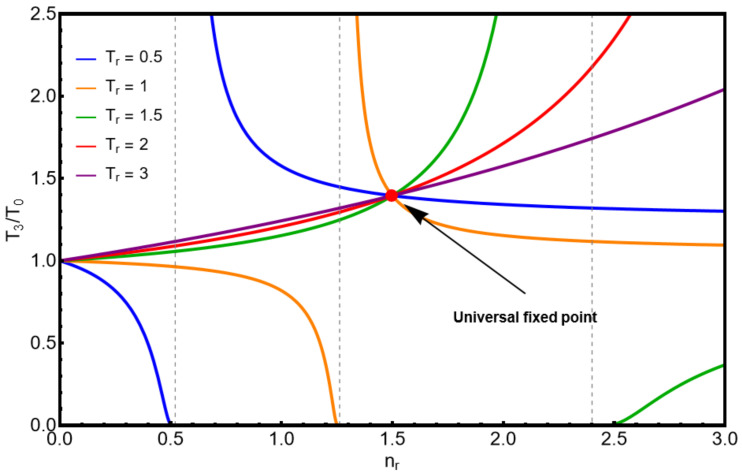
Ratio $T_{3}/T_{0}$, given by Equation (53), as a function of the reduced density $n_{r}$ for several reduced temperatures $T_{r}=0.5,1,1.5,2,$ and 3. All isotherms intersect at the universal fixed point $n_{r}^{*}=3/2$, where ${\left(T_{3}/T_{0}\right)}^{*}=e^{1/3}≃1.3956$. The corresponding pole locations are $n_{r}^{\mathrm{pole}}=0.529,1.263,2.769,5.143$, and 18.0, respectively. The dashed vertical lines indicate these pole positions. The vertical divergences correspond to the pole condition given by Equation (58).

To further illustrate the analytical results derived above, Figure 2 displays the crossover-temperature ratio $T_{3}/T_{0}$, given by Equation (53), as a function of the reduced density $n_{r}$ for several values of the reduced temperature $T_{r}$.

The figure confirms the analytical prediction derived above: all isotherms intersect at the universal fixed point

(56) $$n_{r}^{*}=\frac{3}{2},\;{\left(\frac{T_{3}}{T_{0}}\right)}^{*}=e^{1/3}.$$

This fixed point is the reduced-variable representation of the intrinsic density scale $n^{*}=1/\left(2b\right)$, which emerges directly from the self-consistent crossover relation. Since Equation (53) depends only on the dimensionless variables $n_{r}$ and $T_{r}$, all fluids described within the corresponding-state framework share the same reduced intersection point.

The crossover relation develops singularities whenever its denominator vanishes,

(57) $$1+\frac{n_{r}}{3}-\frac{9}{8}\frac{n_{r}}{T_{r}}=0,$$

which yields the pole location

(58) $$n_{r}^{\mathrm{pole}}=\frac{3T_{r}}{\frac{27}{8}-T_{r}}.$$

These poles correspond to the densities at which the self-consistent denominator vanishes and therefore define the natural boundary of validity of the present mean-field description.

A second characteristic feature follows from the condition

(59) $$\frac{T_{3}}{T_{0}}=1.$$

According to Equation (53), the exponent must vanish, yielding

(60) $$T_{r}=\frac{9}{8}.$$

This special reduced temperature is independent of density and identifies the locus at which the interaction-corrected crossover temperature coincides exactly with the ideal-gas result.

Physically, the fixed point identifies a density at which the influence of attractive and excluded-volume interactions combines in such a way that the crossover-temperature ratio becomes insensitive to temperature. Together, the intrinsic density scale $n^{*}=1/\left(2b\right)$, its universal representation $n_{r}^{*}=3/2$, the pole structure, and the unity condition provide a complete analytical characterization of the crossover-temperature ratio.

## 5. Further Reflections on $T_{3}$ as an Emergent Quantum Threshold

The most interesting aspect is not that quantum effects appear below $T_{3}$. We already know that every classical gas eventually becomes quantum at sufficiently low temperature. The interesting aspect is that

(61) $$T_{3}=T_{3}\left(a,b,m,n\right)$$

emerges internally from the grand-canonical van der Waals (vdW) theory itself. That changes the interpretation considerably. Specifically:

The standard picture:

In most textbooks, the criterion for the breakdown of classical statistics is imposed externally. One introduces the thermal wavelength $\lambda_{T}$ defined in Equation (1) and compares it with the mean intermolecular spacing $n^{-1/3}$. Quantum effects become important when $n\lambda_{T}^{3}∼1$. This criterion comes from quantum statistical mechanics and is not contained in the classical ideal-gas partition function itself. Our van der Waals grand-canonical treatment appears to generate a temperature scale $T_{3}\left(a,b,m,n\right)$ without invoking Bose–Einstein or Fermi–Dirac statistics.

Our theory itself indicates that, *below $T_{3}$, the assumptions underlying the classical vdW description become self-inconsistent*. That is much deeper than simply recovering the thermal-wavelength criterion.

One interpretation is that the grand-canonical van der Waals theory contains enough microscopic information through the particle mass *m*, the excluded volume *b*, and the attractive interaction *a* to estimate its own domain of validity. In other words, the theory is acting as a self-diagnostic model. This logical sequence demonstrates *how the grand-canonical vdW theory endogenously generates the scale $T_{3}$, which in turn establishes the boundary of classical applicability*. Hence, the classical theory predicts where it should stop being trusted.

An emergent quantum threshold:

A stronger interpretation is possible. Notice that *a*, *b*, and *m* encode microscopic physics: (i) *m* fixes the kinetic scale, (ii) *b* fixes the molecular size, and (iii) *a* fixes interaction strength.

The appearance of $T_{3}$ means that these microscopic parameters combine to produce a temperature below which classical fluctuations can no longer sustain a purely classical description.

Thus, $T_{3}$ may be viewed as an emergent quantum threshold generated by the competition between thermal motion and microscopic structure. We ask: when does a system cease to behave classically? Here the answer would be: $T<T_{3}.$ But $T_{3}$ is not imposed by quantum mechanics from outside. It emerges from the fluctuation structure of the van der Waals ensemble itself.

One could therefore argue that *the classical fluctuation structure contains information about the impending breakdown of classical behavior and, consequently, about the approach to the quantum regime*.

## 6. Low-Density Approximation

To gain analytical insight into the competing roles of attractive and repulsive interactions, we examine the behavior of the crossover temperature in the low-density limit, where nb≪1 and βan≪1. Under these conditions, the argument of the exponent in Equation (39) can be expanded to first order in density using a standard Taylor series:

(62) $$\frac{nb-\frac{1}{3}\beta na}{1+nb-\beta an}\approx nb-\frac{1}{3}\beta an.$$

Substituting this linearized argument back into Equation (39) yields the explicit first-order correction for the crossover temperature:

(63) $$T_{3}≃T_{0}\exp\left(nb-\frac{1}{3}\frac{an}{k_{B}T_{0}}\right),$$

where we have approximated the thermal inverse energy in the correction term as $\beta ≃1/(k_{B}T_{0})$, which is valid to first order. Considering $T_{0}$ given by Equation (20) and defining the parameter $\alpha =ame/6\pi \hbar^{2}$, we find

(64) $$\frac{T_{3}}{T_{0}}≃\exp\left(nb-\alpha n^{1/3}\right).$$

In addition, Equation (63) can be written as

(65) $$T_{3}=T_{2}\exp\left(-\frac{1}{3}\frac{an}{k_{B}T_{0}}\right),$$

where $T_{2}=T_{0}\exp\left(2nb/3\right)$ represents the crossover temperature modified exclusively by excluded-volume effects [Equation (28)]. This expression clearly manifests how the quantum crossover temperature is enhanced by particle size considerations nb and suppressed by long-range attractive forces $-an/3k_{B}T_{0}$.

### 6.1. Universal Reduced-Variable Representation

The interaction-corrected crossover temperature obtained in Equation (63) can be rewritten in terms of dimensionless reduced van der Waals variables. Using the critical scaling relations (49) and (50), the density factor scales as

(66) $$n^{1/3}={\left(\frac{n_{r}}{3b}\right)}^{1/3}.$$

Substituting this expression together with $nb=n_{r}/3$ into Equation (63) yields

(67) $$\frac{T_{3}}{T_{0}}=\exp\left(\frac{1}{3}n_{r}-\frac{ame}{2\pi \hbar^{2}}{\left(\frac{n_{r}}{3b}\right)}^{1/3}\right).$$

By defining the dimensionless interaction parameter γ as

(68) $$\gamma =\frac{ame}{2\pi \hbar^{2}{\left(3b\right)}^{1/3}},$$

the temperature ratio assumes a compact universal form:

(69) $$\frac{T_{3}}{T_{0}}=\exp\left(\frac{1}{3}n_{r}-\gamma n_{r}^{1/3}\right)$$

or, in logarithmic form,

(70) $$\ln\left(\frac{T_{3}}{T_{0}}\right)=\frac{1}{3}n_{r}-\gamma n_{r}^{1/3}.$$

This representation clearly separates the competing physical mechanisms:

- The linear term $n_{r}/3$ represents the excluded-volume enhancement.
- The sub-linear term $-\gamma n_{r}^{1/3}$ represents the attractive suppression of the quantum crossover.

The characteristic density scale where these two competing effects balance out is determined by setting the derivative of the logarithm to zero:

(71) $$\frac{d}{dn_{r}}\ln\left(\frac{T_{3}}{T_{0}}\right)=0,$$

which explicitly reads $1/3-\gamma n_{r}^{-2/3}/3=0$, yielding the remarkably clean solution:

(72) $$n_{r}^{*}=\gamma^{3/2}.$$

Thus, the inflection point or minimum that separates the attraction-dominated and repulsion-dominated regimes is entirely controlled by the single dimensionless parameter γ.

**Figure 3:**
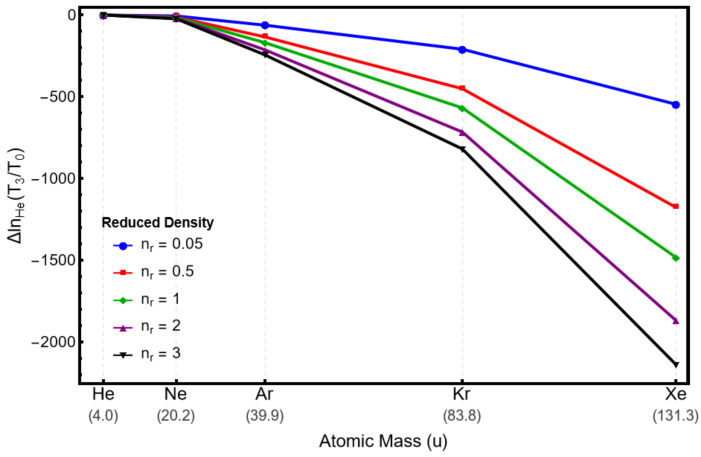
Relative logarithmic ratio $\Delta \ln_{\mathrm{He}}\left(T_{3}/T_{0}\right)$ as a function of atomic mass for the noble-gas family and several fixed reduced densities $n_{r}$. Markers correspond to He, Ne, Ar, Kr, and Xe. The increasingly negative values observed for heavier noble gases reflect the growing interaction-induced displacement of the classical–quantum crossover condition, an effect that becomes stronger as the reduced density increases.

To complement this analysis, Figure 3 illustrates the relative logarithmic ratio normalized to helium for the heavier noble gases (Ne, Ar, Kr, and Xe). By evaluating the curves at specific fixed values of the reduced density $n_{r}$, we can observe how the physical properties scale monotonically with the atomic mass. This multi-fluid comparison highlights that higher atomic masses systematically amplify the attractive deviations from the reference gas, deepening the logarithmic ratio across all selected density isotherms.

**Figure 4:**
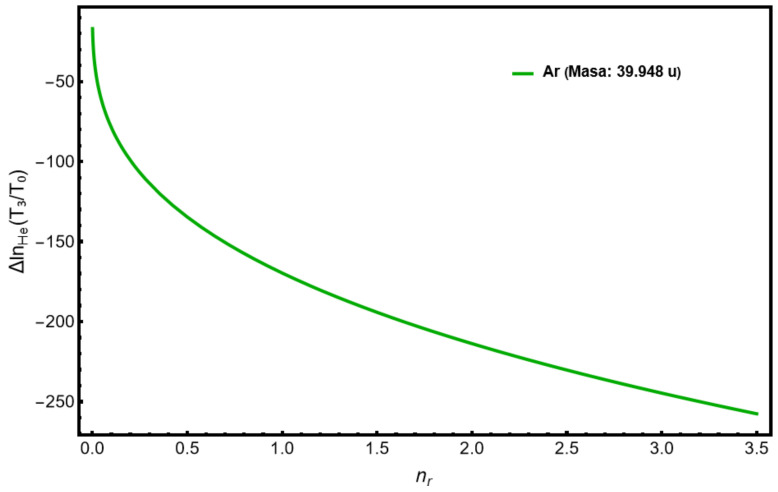
Behavior of the normalized logarithmic ratio for argon (Ar) as a function of the reduced density $n_{r}$. The linear core-repulsion effects cancel out identically, leaving a pure attractive contribution scaling strictly as $n_{r}^{1/3}$.

Furthermore, to isolate the pure effect of the cohesive forces without the geometric constraints, Figure 4 displays the behavior of a single target fluid (argon) when directly subtracted from the helium reference. Because the linear excluded-volume term $n_{r}/3$ cancels out identically in this relative representation, the resulting profile maps the isolated sub-linear attractive scaling factor $\left(\gamma_{\mathrm{He}}-\gamma_{\mathrm{gas}}\right)n_{r}^{1/3}$. The smooth monotonic growth of the curve reflects how the quantum crossover boundary is systematically shifted due to the pure attraction mismatch between the fluids as the system is compressed.

Finally, using the classical van der Waals critical temperature $T_{c}=8a/27bk_{B}$, the parameter γ can be mapped into macroscopic thermodynamic variables as

(73) $$\gamma =\frac{9e}{16\pi }\frac{mk_{B}T_{c}}{\hbar^{2}}{\left(3b\right)}^{2/3}.$$

This definition explicitly connects the quantum-mechanical crossover scale with the classical critical point of the fluid.

### 6.2. Main Findings in the Low-Density Regime

The analytical expressions derived in the low-density limit reveal a rich phenomenology governed by the direct competition between short-range repulsion and long-range attraction. To systematically evaluate how these molecular interactions distort the quantum boundary, we analyze the behavior of the crossover-temperature ratio, $T_{3}/T_{0}$, as a function of the packing fraction nb.

**Figure 5:**
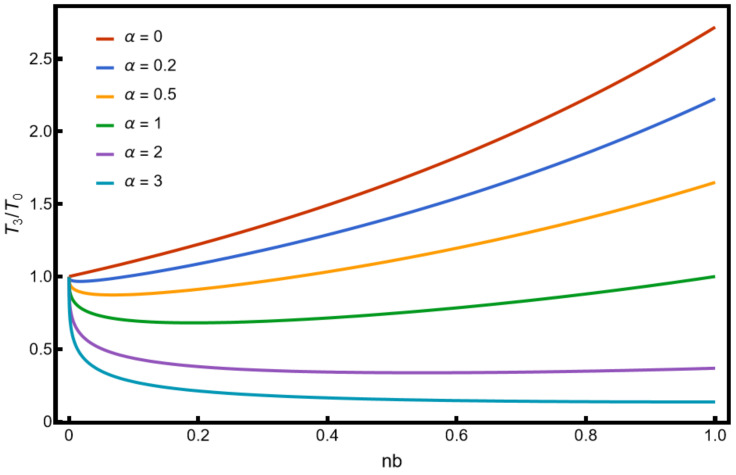
Ratio between the interaction-corrected and ideal crossover temperatures $T_{3}/T_{0}$, given by Equation (64), as a function of the packing fraction nb for several values of the interaction parameter α.

As illustrated in Figure 5, the presence of the attraction parameter α introduces a non-monotonic behavior that drastically departs from the uncorrected ideal-gas scenario. For a purely repulsive system (α=0), the ratio grows exponentially as exp(nb). Physically, this indicates that the spatial crowding induced by the finite size of the particles reduces the available volume in the configuration space, forcing the system to manifest quantum-mechanical characteristics at higher thermal energy scales.

Conversely, when attractive interactions are turned on (α>0), a distinctive localized drop occurs in the low-density regime. Near the origin (n→0), the sub-linear scaling of the attractive contribution, which varies as $n^{1/3}$, possesses a mathematically infinite derivative with respect to density. Consequently, cohesive intermolecular forces initially dominate over the linear excluded-volume enhancement. This mutual braking mechanism among particles lowers their effective kinetic energy, thereby shielding the system from quantum effects and extending the domain of validity of the classical Maxwell–Boltzmann description toward lower temperatures.

As the density increases further, the linear term eventually overcomes the sub-linear attractive suppression, leading to the characteristic minimum predicted by Equation (71) before entering the repulsion-dominated exponential ascent.

**Figure 6:**
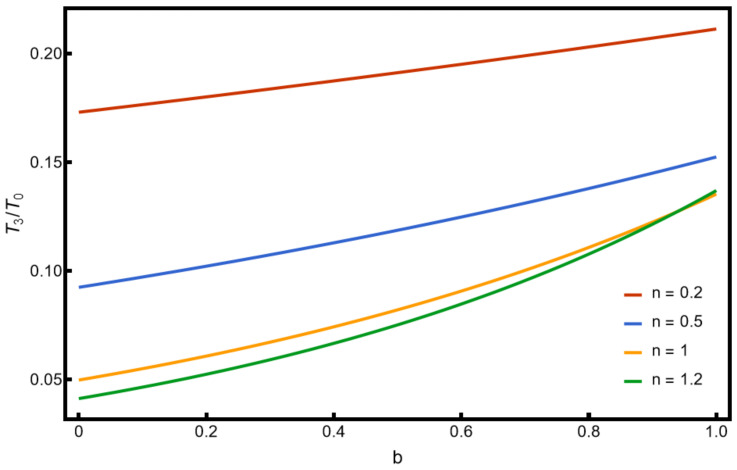
Ratio between the interacting and ideal crossover temperatures $T_{3}/T_{0}$, given by Equation (64), as a function of *b* for several values of the density n=0.2,0.5,1,1.2, with interaction parameter α=3.

In Figure 6 we display the behavior of the ratio between the interacting and ideal crossover temperatures, $T_{3}/T_{0}$, as a function of the excluded-volume parameter *b* for several values of the density *n*. The figure shows that the crossover temperature increases monotonically with *b*, showing that excluded-volume effects favor the emergence of quantum behavior at higher temperatures. Moreover, the effect becomes more pronounced as the density increases since excluded-volume corrections scale with the product nb.

**Figure 7:**
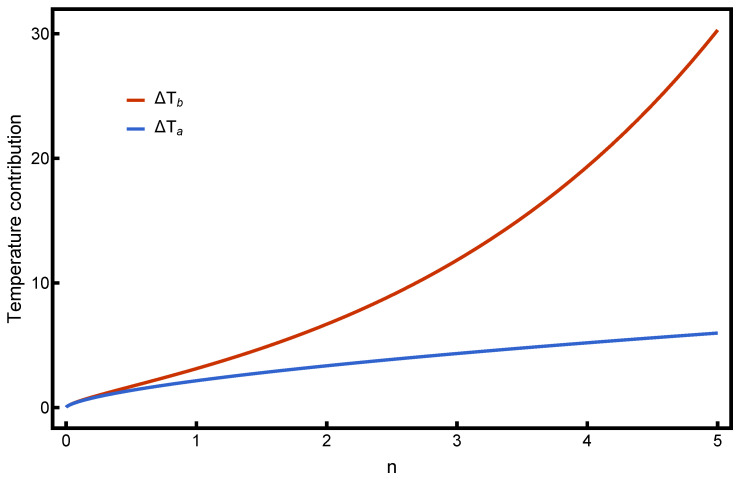
Separate contributions of excluded-volume and attractive interactions to the crossover temperature versus density *n*. The repulsive branch increases with density, while the attractive contribution systematically lowers the crossover scale. Here, we take a=0.5, b=0.3, m=1, $k_{B}=1,\;$ℏ=1.

In order to separately visualize the effects of repulsive and attractive interactions on the crossover temperature, we plot in Figure 7, as functions of the density *n*, the quantities

(74) $$\Delta T_{b}=T_{0}\exp\left(nb\right),$$

associated with excluded-volume repulsion, and

(75) $$\Delta T_{a}=T_{0}\exp\left(-\alpha n^{1/3}\right),$$

associated with long-range attractive interactions.

The figure clearly illustrates the physical competition between both mechanisms. The repulsive contribution $\Delta T_{b}$ monotonically increases the crossover temperature, shifting the onset of quantum-mechanical behavior toward higher temperatures due to spatial crowding effects. Conversely, the attractive contribution $\Delta T_{a}$ lowers the crossover scale, thereby extending the domain of validity of the classical Maxwell–Boltzmann regime through cohesive intermolecular correlations.

**Figure 8:**
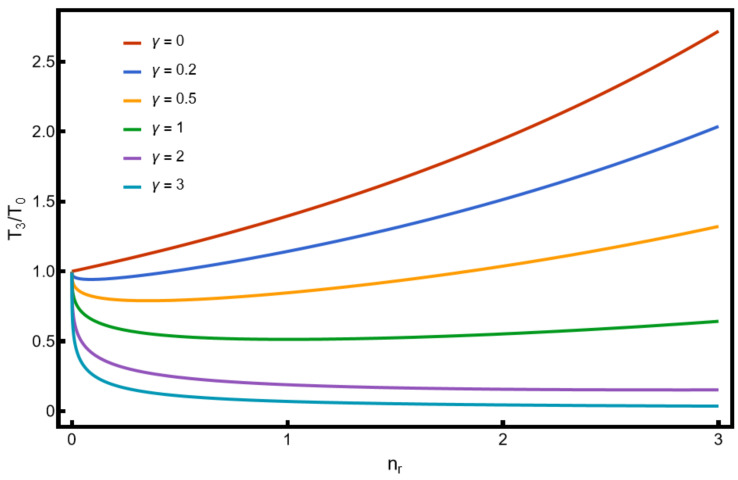
Interaction-corrected crossover-temperature ratio $T_{3}/T_{0}$, given by Equation (69), as a function of the reduced density $n_{r}$ for several values of the dimensionless interaction parameter γ.

In Figure 8, we plot the ratio between the interaction-corrected and ideal crossover temperatures, $T_{3}/T_{0}$, given by the universal scaling function in Equation (69).

By mapping the system into reduced van der Waals variables, the behavior of all possible fluids collapses onto a single family of curves parameterized solely by the dimensionless interaction parameter γ. This representation highlights the universality of the quantum crossover: the structural topology of the thermodynamic boundary—including the exact location of the minimum $n_{r}^{*}=\gamma^{3/2}$—depends exclusively on the ratio between the classical critical scale ($T_{c}$, *b*) and the quantum confinement scale ($\hbar^{2}/m$). For higher values of γ, the attraction-dominated “valley” deepens and extends further into higher-density regimes, shifting the recovery point toward larger reduced densities.

**Figure 9:**
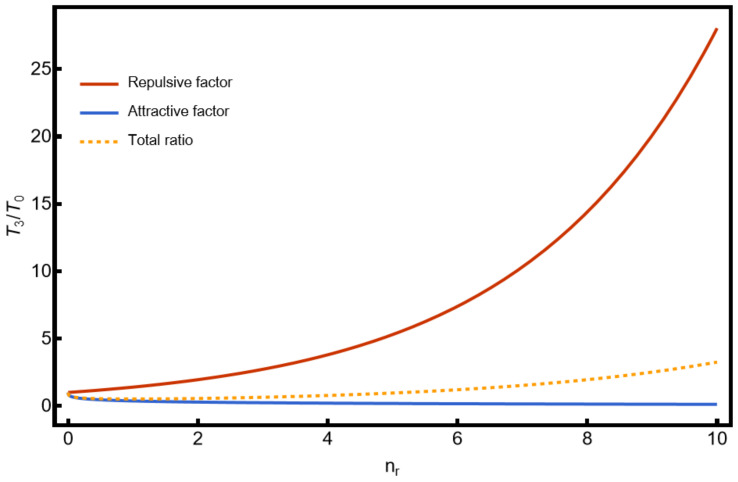
Separate contributions of excluded-volume and attractive interactions to the crossover temperature in the reduced-variable representation given by Equation (69). The linear repulsive branch increases with reduced density, while the sub-linear attractive contribution lowers the crossover scale.

In Figure 9, we inspect the decoupled physical mechanisms in this dimensionless domain by plotting the separate contributions of excluded-volume and attractive interactions as functions of the reduced density $n_{r}$. Specifically, we display the repulsive branch $\exp(n_{r}/3)$ alongside the attractive scaling factor $\exp(-\gamma n_{r}^{1/3})$.

The curves visually demonstrate how the sub-linear attractive contribution dominates the low-density landscape due to its steep initial slope, pull-down effect, and spatial reach. As the fluid is compressed past the characteristic scale $n_{r}^{*}$, the linear packing contribution grows exponentially faster, eventually forcing the total crossover temperature to reflect the core-repulsion physics of a dense fluid. This reduced-variable analysis confirms that the macroscopic manifestation of the quantum crossover boundary is a direct consequence of a microscopically scaled competition between cohesive and steric molecular correlations.

## 7. Conclusions

In this work we have investigated the classical–quantum crossover in an interacting dilute van der Waals fluid within a self-consistent grand-canonical mean-field framework. Starting from an interaction-corrected description of the density fluctuations, we derived an explicit crossover temperature $T_{3}\left(a,b,m,n\right)$ that depends on the particle mass, density, and microscopic van der Waals parameters characterizing excluded-volume and attractive interactions.

The resulting crossover scale preserves the familiar ideal-gas dependence $T\propto n^{2/3}$ while acquiring nontrivial interaction corrections. Excluded-volume effects systematically increase the crossover temperature, favoring the onset of the quantum-degenerate regime at higher temperatures, whereas attractive interactions produce an opposite tendency, lowering the crossover scale and extending the apparent domain of validity of the classical Maxwell–Boltzmann description. Within the low-density regime, the leading repulsive and attractive corrections scale as *n* and $n^{1/3}$, respectively.

A particularly remarkable result emerges when the theory is expressed in reduced van der Waals variables. The complete crossover-temperature ratio $T_{3}/T_{0}$ exhibits a universal fixed point located at

$$n_{r}^{*}=\frac{3}{2},\;{\left(\frac{T_{3}}{T_{0}}\right)}^{*}=e^{1/3}.$$

This fixed point is independent of temperature and of the specific microscopic parameters of the fluid. The appearance of this invariant point demonstrates that the crossover structure possesses a universal component that survives the reduction to corresponding-state variables. In addition, the theory predicts a well-defined pole structure associated with the vanishing of the self-consistent denominator, thereby identifying natural boundaries for the validity of the dilute mean-field description.

Beyond these quantitative results, the most significant outcome of the present analysis is conceptual. In standard treatments, the onset of quantum degeneracy is characterized through the thermal de Broglie wavelength criterion. In the present approach, this criterion is complemented by a self-consistent grand-canonical treatment of the van der Waals fluid, which leads to an interaction-corrected crossover temperature $T_{3}$. Although the thermal de Broglie wavelength introduces the quantum crossover scale, the interaction-induced correction is obtained without making explicit use of the Bose–Einstein or Fermi–Dirac distribution functions. The resulting temperature defines an interaction-corrected boundary of validity of the classical van der Waals description.

This observation provides a complementary perspective on the classical–quantum crossover. Rather than replacing the conventional thermal-wavelength criterion, the present approach shows how intermolecular interactions modify the crossover temperature through a self-consistent grand-canonical treatment. In this sense, the van der Waals grand-canonical ensemble acts as a self-consistent framework for estimating the interaction-corrected boundary of validity of the classical description.

The interaction-corrected crossover temperature $T_{3}$ suggests that classical interacting-fluid theories may contain valuable information about the limits of their own applicability when combined with the standard quantum-degeneracy criterion. Whether this interaction-induced correction persists beyond the mean-field approximation and extends to more realistic interacting fluids remains an open question. Future work will explore possible connections with information-theoretic measures, thermodynamic geometry, Fisher-information approaches, and complexity-based indicators of classical–quantum crossover.

## Figures and Tables

**Table 1:** Comparison of the characteristic temperature scales for ^4^He at the liquid density $\rho =125\;\mathrm{kg}\;m^{-3}$.

Temperature	Value (K)	Physical Significance
$T_{0}$	5.39	Ideal-gas crossover temperature
$T_{3}$	2.63	Interaction-corrected crossover (this work)
$T_{\mathrm{BEC}}$	3.13	Bose–Einstein condensation (ideal Bose gas)
$T_{\lambda }$	2.17	Superfluid transition of ^4^He

## Data Availability

Data are contained within the article.
